# An Extraordinary Case of Distension of the Œsophagus, Forming a Sac, Extending from Two Inches below the Pharynx to the Cardiac Orifice of the Stomach

**Published:** 1821-12

**Authors:** T. Purton

**Affiliations:** Member of the Royal College of Surgeons.


### *An extraordinary Case of Distension of the Œsophagus, forming a Sac, extending from two inches below the Pharynx to the Cardiac Orifice of the Stomach.* By T. PURTON, F.L.S. and Member of the Royal College of Surgeons.

ALTHOUGH the œsophagus is subject to but few diseases, and those, for the most part, simple in their nature, yet are its functions often impeded by obstructions which produce a train of symptoms truly distressing, and often fatal. Many such cases, from indurated glands compressing, from without, some part of the tube, have come under my care:* others, the

---

* Two fatal cases of this kind have occurred in my own practice: the subjects of them were married women, of rather an advanced age, who were afflicted with enlarged glandular tumors seated beneath the upper part of the sternum, which pressed not only on the œsophagus, but also on the trachea, and to that degree consequence of extraneous substances having passed into the canal,—such as pins, needles, corks, and even sponge. The present case, however, of more than twenty years' standing, I believe to differ from any one yet recorded; at least, any that has come under my notice.

The history of the case is short. John Osborne, a blacksmith at Broom, aged 43, when a youth, received a severe blow on the sternum, which deprived him for some minutes of sense and motion; ever since which time he has laboured under more or less difficulty in swallowing. Throughout the last twenty years, during which time I have occasionally attended him, he has suffered, at intervals, many very severe attacks, continuing sometimes for three weeks or more; during the whole of this time scarcely any food entering the stomach. Occasionally, however, he was able, for months together, to propel, after violent exertions, the contents of the sac into the stomach. If the food was not propelled into the stomach by a certain degree of force, he would reject it; so that latterly he never attempted to use any violent efforts, but would suffer it to remain in the sac for hours, and even days: and probably it was in part digested, as the internal surface of the sac presented, in a slight degree, the rugose appearance of the stomach. An attack would often be induced by irritation of mind, or by a constipated state of the bowels: in this latter case, he was often relieved by emptying the rectum by means of a stimulating enema. No kind of food passed through the contracted cardia, until the sac above it was quite distended; nor did he, until then, attempt to use any voluntary exertion, as experience had taught him that previous efforts were ineffectual. For the last three months he was confined to the house, and for six weeks to his bed; during the whole of which time he was able to pass little or no food beyond the sac, but was sustained by the constant use of nourishing injections.

On dissection, the œsophagus was found greatly distended, forming a sac, or pouch, reaching from within two inches of the pharynx to the cardia: it contained, by admeasurement, full two quarts. The cardiac orifice was found pervious, but much contracted: no particular appearance of disease was remarked in any other viscus. The liver, however, was paler than usual; but the stomach was perfectly healthy. On the posterior part of the sac, putrefaction had commenced; and, on filling it, this part gave way.

The poor sufferer had been examined by Sir Astley Paston Cooper and Mr. Freer of Birmingham; also by most of the medical men in this neighbourhood; but he derived no benefit

---

that, in their first labours, I fully expected they would be strangled before the children could be born; yet, after all my anxiety, although safely delivered, they both died in a few days.

from the treatment recommended. I made out, to my own satisfaction, the nature of the disease some years ago, by passing the common probang into the stomach; which I did with much difficulty, as it could not be an easy matter to find so small an orifice in so wide a canal, especially as the sac must have formed a kind of *fosse* around it. On endeavouring to withdraw the probang, I was a good deal alarmed by the degree of force by which it was detained, fearing lest the sponge might be left in the stomach, or that the cardiac orifice or some part of the canal might be lacerated: by gradual, but considerable, expulsive force being used, it was at length withdrawn, and, on its passing the orifice, it made a report so loud as to alarm the bystanders.

Sir A. P. Cooper recommended the trial of bougies; but, from the difficulty of introducing them, and the great pain and irritation consequent upon their use, they were laid aside: since then only palliative measures have been had recourse to.

I have made a preparation of the stomach and the sac-like œsophagus, which I intend to present to the College of Surgeons, should they deem it worthy of a place in their museum.

*Alcester; Oct. 13th,* 1821.

